# Reverse Microbiomics: A New Reverse Dysbiosis Analysis Strategy and Its Usage in Prediction of Autoantigens and Virulent Factors in Dysbiotic Gut Microbiomes From Rheumatoid Arthritis Patients

**DOI:** 10.3389/fmicb.2021.633732

**Published:** 2021-02-25

**Authors:** Haihe Wang, Edison Ong, John Y. Kao, Duxin Sun, Yongqun He

**Affiliations:** ^1^Department of Pathogen Biology, Harbin Medical University (Daqing), Daqing, China; ^2^Unit for Laboratory Animal Medicine, University of Michigan, Ann Arbor, MI, United States; ^3^Department of Computational Medicine and Bioinformatics, University of Michigan Medical School, Ann Arbor, MI, United States; ^4^Department of Internal Medicine, Division of Gastroenterology and Hepatology, University of Michigan Medical School, Ann Arbor, MI, United States; ^5^Department of Pharmaceutical Sciences, College of Pharmacy, University of Michigan, Ann Arbor, MI, United States; ^6^Department of Microbiology and Immunology, University of Michigan Medical School, Ann Arbor, MI, United States; ^7^Center of Computational Medicine and Bioinformatics, University of Michigan, Ann Arbor, MI, United States

**Keywords:** reverse microbiomics, reverse vaccinology, microbiome, gut microbiota, rheumatoid arthritis, ontology, bioinformatics

## Abstract

Alterations in the gut microbiome have been associated with various human diseases. Most existing gut microbiome studies stopped at the stage of identifying microbial alterations between diseased or healthy conditions. As inspired by reverse vaccinology (RV), we developed a new strategy called Reverse Microbiomics (RM) that turns this process around: based on the identified microbial alternations, reverse-predicting the molecular mechanisms underlying the disease and microbial alternations. Our RM methodology starts by identifying significantly altered microbiota profiles, performing bioinformatics analysis on the proteomes of the microbiota identified, and finally predicting potential virulence or protective factors relevant to a microbiome-associated disease. As a use case study, this reverse methodology was applied to study the molecular pathogenesis of rheumatoid arthritis (RA), a common autoimmune and inflammatory disease. Those bacteria differentially associated with RA were first identified and annotated from published data and then modeled and classified using the Ontology of Host-Microbiome Interactions (OHMI). Our study identified 14 species increased and 9 species depleted in the gut microbiota of RA patients. Vaxign was used to comparatively analyze 15 genome sequences of the two pairs of species: Gram-negative *Prevotella copri* (increased) and *Prevotella histicola* (depleted), as well as Gram-positive *Bifidobacterium dentium* (increased) and *Bifidobacterium bifidum* (depleted). In total, 21 auto-antigens were predicted to be related to RA, and five of them were previously reported to be associated with RA with experimental evidence. Furthermore, we identified 94 potential adhesive virulence factors including 24 microbial ABC transporters. While eukaryotic ABC transporters are key RA diagnosis markers and drug targets, we identified, for the first-time, RA-associated microbial ABC transporters and provided a novel hypothesis of RA pathogenesis. Our study showed that RM, by broadening the scope of RV, is a novel and effective strategy to study from bacterial level to molecular level factors and gain further insight into how these factors possibly contribute to the development of microbial alterations under specific diseases.

## Introduction

Microbiota are now widely recognized as the key player in maintaining host health. They have a profound impact on human diseases, including obesity (Baothman et al., 2016), diabetes (Alkanani et al., 2015), cardiovascular diseases (Tang et al., 2017), inflammatory bowel diseases, cancers (Lucas et al., 2017), and autoimmune diseases such as rheumatoid arthritis (Holmdahl et al., 2014). Dysbiosis represents an altered balance of protective and detrimental commensals that are associated with disease development. It is frequently characterized by enrichment or depletion of bacteria or fungi (Forbes et al., 2018; Huh and Roh, 2020). Recent microbiome studies have detected many bacterial alterations in various host conditions, which might contribute to the formation of dysbiosis (Logan et al., 2016; Colquhoun et al., 2020). However, it remains a huge challenge to systematically analyze and convert microbiome data into meaningful biological insights.

Rheumatoid arthritis (RA) is one of the most prevalent chronic inflammatory diseases, characterized by inflammation in the connecting or supporting structures of the body (Bax et al., 2011; Smolen et al., 2016). RA is thought to be initiated by an infection that may affect immune tolerance or provide an antigen that mimics a host protein, but the exact mechanisms of RA are still unclear (Smolen et al., 2016). It is estimated that 50% ∼ 80% of RA patients possess auto-antibodies (Derksen et al., 2017). The auto-antigens and other microbial factors may stimulate autoimmune inflammatory responses involving auto-antibody-producing B cells and the release of various pro-inflammatory cytokines such as TNF-α (Rosenthal et al., 2015). Emerging evidence indicates that human gut microbiota may play a critical role in the pathogenesis of RA (Zhang et al., 2015).

In patients with inflammatory arthritis, the degradation products of cell walls and the nucleic acids of intestinal bacteria are detected in the inflamed joints (Van Der Heijden et al., 2000; Schrijver et al., 2001). Immunoglobulin A (IgA) anti-citrullinated protein antibody (ACPA) is detectable before the onset of arthritis (Rantapaa-Dahlqvist et al., 2003), suggesting that RA originates at mucosal sites such as the gut and oral cavity. A recent mouse study showed that gut dysbiosis contributes to arthritis development via the activation of auto-reactive T cells in the intestine (Maeda et al., 2016). The specific microbial alterations in the human guts have been suggested as potential ways for the prognosis and diagnosis of RA (Maeda and Takeda, 2019; Lee, 2020). *Prevotella copri* and *Prevotella histicola* have been found to be associated with RA (Scher et al., 2013; Maeda and Takeda, 2017). *P. copri* is an obligately anaerobic, non-spore-forming Gram-negative bacterium. The abundance of *P. copri* was elevated in untreated recent-onset RA patients and reduced in patients with chronic RA, psoriatic arthritis, and healthy volunteers (Scher et al., 2013). In contrast, *P. histicola* isolated from the commensal bacteria in the human gut was able to reduce the severity of collagen-induced arthritis in HLA-DQ8 mice (Maeda and Takeda, 2017).

Increasing research interest is to understand how microbiomes influence or influenced by the changes in host physiology, health, and even lifestyle. To address these questions, some studies have been performed to characterize the composition of host-associated microbiomes. The traditional culture-based approach can only be applied on a small scale and to relatively well-understood biological systems, and very few (<1%) microbial organisms can be cultured in a laboratory (Hugenholtz et al., 1998). Next-generation sequencing-based metagenomic and community genomics approaches have allowed researchers to investigate the genomes of all species present in a given complex environment. Today there are many other technologies for analyzing microbial communities, including shotgun metagenomics, 16S rDNA sequencing, or the more classical PCR. The Metagenome-Wide Association Study (MWAS) is a method that applies a metagenomics shotgun sequencing method to identify the associations between the microbiome and diseases (Wang and Jia, 2016).

Most current microbiota and dysbiosis studies ended at the stage of identifying new microbes differentially enriched (increased) or depleted (decreased) in dysbiotic patients compared to healthy controls. Such studies are useful in uncovering microbial profiles in microbiome-associated diseases. However, given more microbiome profiles identified, it has become urgent, and yet a big challenge, to further study the underlying gene-level pathogenesis mechanisms that contribute to the diseases, which would lead to preventative and therapeutic measures against diseases. To address the challenge of efficiently identifying the underlying molecular mechanisms of microbiome-related diseases, we propose a Reverse Microbiomics (RM) strategy.

The proposed RM strategy is inspired by Reverse Vaccinology (RV), which is an emerging vaccine development methodology that predicts vaccine targets by bioinformatics analysis of pathogen genomes (Rappuoli, 2000). RV facilitates rapid vaccine design by utilizing bioinformatics analysis to prioritize promising vaccine candidates and reduce the resources required in conventional animal testing and clinical trials. RV successfully has been applied to the development of vaccines against a variety of pathogens such as *Neisseria meningitidis* serogroup B (MenB) (Pizza et al., 2000), *Streptococcus pneumoniae* (Wizemann et al., 2001), and *Bacillus anthracis* (Ariel et al., 2002). The Vaxign program is the first web-based RV program, developed by us, which has been widely used for RV vaccine target prediction and bacterial genome annotations (He et al., 2010b; Ong et al., 2017). We have recently further extended the capabilities of Vaxign by incorporating machine learning and developed Vaxign-ML, which improves the prediction accuracy and shows superior performance compared to other existing open-source RV tools (Ong et al., 2020). Anticipating that RV methods and tools may be extended to research other than vaccinology, RM employs both the methods and tools of RV. The RM methodology proceeds in the following manner: First, the microbial alterations associated with a specific disease or dysbiosis are identified. RM then proceeds with the ontology-based annotation and classification of enriched or depleted bacteria. Finally, we perform comparative pan-genome sequence-based bioinformatic analysis of these dysbiotic microbiota to rapidly identify auto-antigens or virulence factors contributing to diseases. The proposed RM pipeline provides more rapid identification of significant microbiota for dysbiosis studies to facilitate more resource-efficient animal testing and clinical trials.

In this study, we describe the development of the RM strategy and apply this strategy to the systematic identification of microbial gene factors contributing to the pathogenicity of RA. We found 23 gut bacteria species that played an important role in the disease of RA. We selected four of these species through our ontology-based comparative analysis that were grouped into two pairs (Gram-negative *Prevotella copri* and *Prevotella histicola*, and Gram-positive *Bifidobacterium dentium* and *Bifidobacterium bifidum*). After analyzing 15 genome sequences of these two gut bacteria pairs, we successfully predicted and evaluated many auto-antigens and potential virulence factors that are likely to be associated with RA.

## Materials and Methods

### A Meta-Analysis of RA-Related Gut Microbiota Profiles

RA related microbiome literature was searched on PubMed with the keywords “microbiome,” “microbiota,” and “Rheumatoid arthritis.” Each up- or down-regulated bacterium to be included in the study required that the bacterial alteration in the RA-associated microbiome must be concluded from the original peer-reviewed experiment-based research paper(s). Review articles were also used as sources for original research papers. Experimental assays identifying differential bacterial profiles included traditional culture-based approaches, 16S rRNA sequencing, and metagenomic sequencing. The meta-analysis identified enriched (increased) or depleted (decreased) microbe in RA patients compared to healthy controls. Only those enriched (increased) or depleted (decreased) microbe in RA patients compared to healthy controls with significant statistics (*p*-value < 0.05) were included in the next analysis. The annotated differentially regulated bacteria, taxonomy ID, rank, location, PMID, and description information were collected in a pre-defined Excel file.

### Ontological Classification of Altered Microbes in RA-Related Gut Microbiota

All the differentially regulated bacteria were all mapped to the corresponding terms in the NCBI Taxonomy ontology (NCBITaxon). The Ontofox tool (Xiang et al., 2010) was then used to extract the bacterial terms and their associated ancestors in NCBITaxon in a hierarchical structure. Such information was also ontologically annotated and represented in the Ontology of Host-Microbiome Interactions (OHMI) as previously described (He et al., 2019). Protégé OWL-editor (Musen, 2015) was used for ontology edit and visualization.

### Genome Sequence Extraction and Vaxign Analysis

While different taxonomic levels (e.g., species, genus, and family) of enriched or depleted bacteria were found to be differentially regulated, only those bacteria altered at the species level were considered for the next step analysis due to the feasibility of identifying genomes matching these species. To make the prediction more specific, we selected pairs of bacterial species, and each pair consisted of enriched and depleted bacteria. With these selection criteria, we finally chose two pairs of representative bacterial species for further comparative genomics analysis, including (i) *Prevotella copri* (enriched) vs. *Prevotella histicola* (depleted); (ii) and *Bifidobacterium dentium* (enriched) vs. *Bifidobacterium bifidum* (depleted). The genome sequences of these four species were retrieved from the NCBI database. In addition, the information of these genomes, including strain names, NCBI BioProject numbers, NCBI BioSample numbers, and protein size, were also retrieved and summarized in Excel. All completed and annotated genomes with their NCBI BioProject numbers were used for Vaxign dynamic analysis. Given the BioProject numbers, Vaxign automatically retrieved the protein sequences of the chromosome and any possible plasmid(s) of these bacteria. For each protein, the Vaxign pipeline was used to compute various features, including subcellular localization, conservation among different strains, exclusion from non-pathogenic strains, and homology with human proteins (He et al., 2010b; Ong et al., 2017). The default Vaxign settings were applied in our analyses. For example, the adhesin probability cutoff of 0.51 was used for adhesin prediction.

### Protective Antigenicity Prediction

Vaxign-ML was used to calculate and rank the corresponding protective antigenicity (protegenicity) scores of potentially pathogenic bacterial antigens (Ong et al., 2020). Vaxign-ML is a machine learning (ML)-based vaccine candidate prediction system based on the extreme Gradient Boosting model. Vaxign-ML utilized biological and physicochemical features computed from each of the protein sequences as the input of the ML model. The nested five-fold cross-validation (N5CV) and leave-one-pathogen-out (LOPOV) validation were used to ensure unbiased performance assessment and the capability to predict vaccine candidates for a new emerging pathogen. The output of Vaxign-ML is the percentile rank score as described by Ong et al. (2020). A score over 90% is considered as a predicted protective antigen that has strong antigenicity and likely induces protective immunity when used as a vaccine antigen.

### RM Software Availability and Implementation

The RM analysis pipeline reported in this article includes several software tools. The Ontobee tool for ontology term search is available at http://www.ontobee.org/. The Ontofox tool for taxonomy subclass extraction is available at http://ontofox.hegroup.org/. The Protégé-OWL editor for ontology visualization and editing can be downloaded from https://protege.stanford.edu/. The Vaxign program for comparative genome analysis is available at http://www.violinet.org/vaxign. The Vaxign-ML machine learning tool is available at http://www.violinet.org/vaxign/vaxign-ml. The Docker standalone Vaxign-ML is available at https://hub.docker.com/r/e4ong1031/vaxign-ml. The source code of the Vaxign-ML is available at https://github.com/VIOLINet/Vaxign-ML-docker (He et al., 2010b; Ong et al., 2017).

### COG Functional Annotation and Multiple Sequence Alignment

The COG protein function assignment was performed using the EggNOG 4.5 server (http://eggnogdb.embl.de/#/app/home).

### Evaluation of Predicted Results

The predicted virulence factors or auto-antigens from our RM method were compared with experimentally identified results, which were shown to contribute to the pathogenesis of autoimmune for RA or other related rheumatic diseases in the literature. However, only a portion of our predictions was verified by this approach. The remaining unverified predictions were considered as new predictions and were subjected to experimental verification. Hence, our RM study illustrates its usefulness in generating new hypotheses by incorporating all related results, literature results, and logical justification.

## Results

### Summary of the Proposed Reverse Microbiomics (RM) Strategy

**FIGURE 1:**
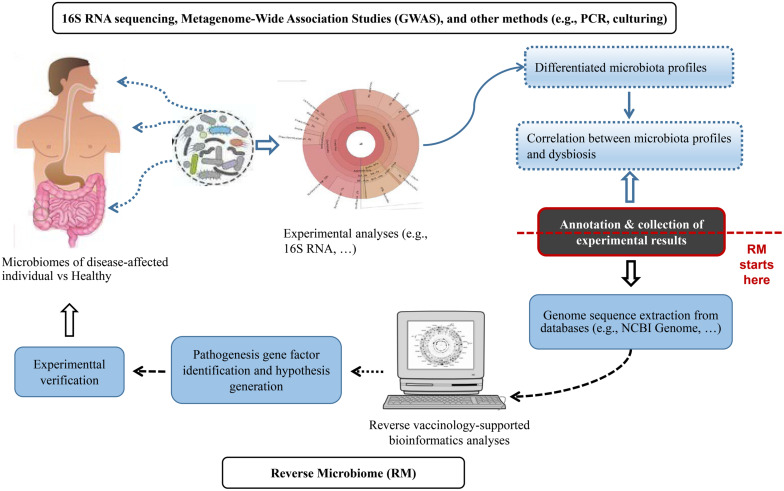
The Reverse Microbiomics (RM) strategy. RM starts from the collection and meta-annotation of experimental results of differentially changed microbial profiles associated with a specific disease or dysbiosis. Genome sequences can be extracted from NCBI Genome or other databases and analyzed to identify microbial gene factors that contribute to disease pathogenesis. New hypotheses may also be generated and experimentally verified.

Figure 1 summarized the RM strategy. The proposed RM strategy started with the bioinformatical collection and analysis of microbiome profiles from published experimental studies. The published results included the enrichment or depletion of microbes at different levels of the taxonomy (e.g., species, genus, and family, etc.) under dysbiotic conditions. We proposed the usage of a hierarchical taxonomy relation extraction tool, Ontofox (Xiang et al., 2010), to extract and identify the relations among microbes in a hierarchical structure. Next, we retrieved genome sequences from the NCBI Genome Project. Vaxign was then applied to perform comparative sequence analysis and identify genes that were conserved in enriched bacteria but not present in depleted bacteria. We found that it was more effective to have protein sequences from more than one genome, ideally at least one from enriched and another from depleted bacteria species in the disease-associated samples. We could then identify auto-antigens by comparing the bacterial protein sequences with human proteins. The analysis of the identified microbial genes from RM would generate novel hypotheses subjected to experimental verification (Figure 1).

In RV, protective bacterial antigens suitable for vaccine development are typical outer membrane proteins, secreted proteins, and adhesin proteins (He et al., 2010a; Ong et al., 2017). These proteins are also often virulence factors since they are more likely to have direct contact with the host cells or the extracellular environment, and thus are more susceptible to induce host humoral and cell-mediated responses. We hypothesized that these proteins were potential virulence factors for microbiome-associated diseases such as RA. Our RM methodology was specifically applied to address this hypothesis.

### Differentiated Bacterial Profiles in Gut Microbiomes From RA Patients

**FIGURE 2:**
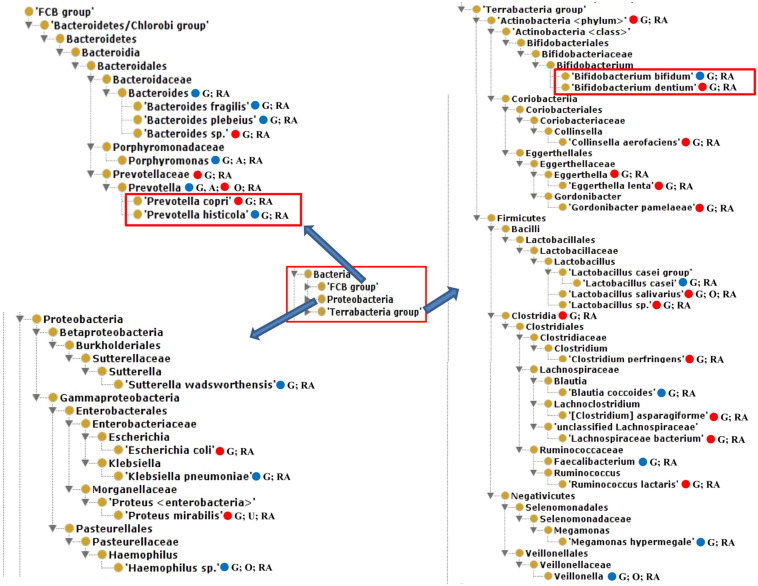
The hierarchical structure of 23 significantly changed bacteria from the guts of rheumatoid arthritis (RA) patients. In addition to their presence in the gut of RA patients, some of these bacteria were also found in oral or urine locations and possibly in ankylosing spondylitis (AS) patients. Color meanings: Red – increased species; Blue – decreased species. G, Gut; O, oral; U, urine.

Our literature mining and manual annotation of over 20 peer-reviewed publications (Supplementary Table 1) identified 54 bacteria that were statistically differentially enriched or depleted in the gut, oral, airway, and urine samples of RA patients compared with healthy and pre-defined human subjects (Supplementary Table 2). These 54 bacteria were located at different taxonomy levels. Out of these 54 bacteria, 23 were at the level of species, among which 14 were enriched and 9 were depleted in RA patients. Figure 2 displayed the hierarchical structure of these 23 species in an ontological representation. As shown in Figure 2, four bacterial species were enriched under the order of *Clostridiales*, including *Clostridium perfringens*, *Clostridium asparagiforme*, *Lachnospiraceae bacterium*, and *Ruminococcus lactaris*. Similarly, two bacterial species under the family of *Eggerthellaceae*, including *Eggerthella lenta* and *Gordonibacter pamelaeae*, were enriched. *Bacteroides plebeius, Bifidobacterium bifidum*, *Lactobacillus casei*, and *Prevotella histicola* were found to be significantly depleted in the guts of RA patients. In contrast, *Bacteroides* sp., *Bifidobacterium dentium*, *Lactobacillus salivarius*, *Lactobacillu*s sp., *and Prevotella copri* were significantly enriched in the guts of RA patients.

### Differentiated RA-Related Microbial Genes by RM Analysis

**FIGURE 3:**
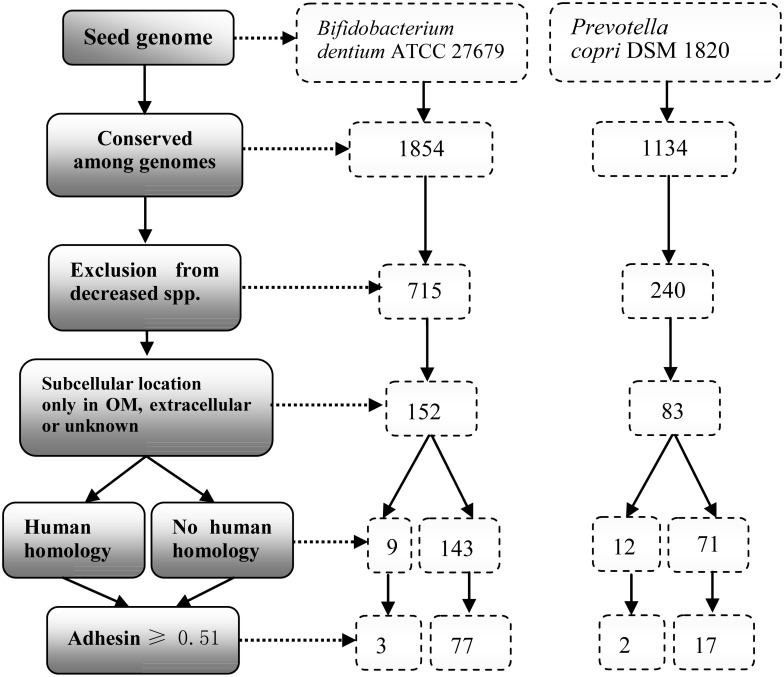
Vaxign analysis workflow and results. The genome of *B. dentium* strain ATCC 27679 was used as the seed genome for the analysis. Among proteins from 4 *B. dentium* genomes, 1,854 proteins were conserved. By excluding proteins found in 6 *B. bifidum* genomes, there were 715 *B. dentium* proteins remained, from which, 152 were identified to be in the outer membrane (OM), extracellular, or unknown locations. Among these 152 proteins, 9 have homology with human proteins, and 3 of these 9 proteins were predicted to have adhesin probabilities ≥ 0.51. On the other hand, there were 77 proteins predicted as adhesins with no human homology. In the group of *Prevotella, P. copri* DSM 1820 is used as a seed genome. With the same workflow and criteria, we obtained 12 and 71 proteins with and without human homology, respectively. Among these 83 proteins, Vaxign analysis identified 2 adhesins with human homology while 17 adhesins had no human homology.

We hypothesized that specific pathogenic virulence factors were commonly found in the pathogenic (enriched) strains, which were also rarely found in probiotic (depleted) strains in the microbiome-associated diseases. To demonstrate the usage of the RM strategy, we selected two pairs of representative bacterial species (*P. copri* vs. *P. histicola* and *B. dentium* vs. *B. bifidum*). The first pairs were the *P. copri and P. histicola*, which were enriched and depleted in RA patients, respectively. *P. copri* has been known to play an important role in influencing the pathogenesis of RA (Scher et al., 2013) (Supplementary Table 1). Another pair included *B. dentium* and *B. bifidum*. Intestinal *B. dentium* expansion strongly correlates with enhanced susceptibility to untreated RA, and the increase in *B. dentium* abundance is accompanied by a reduction in *B. bifidum* (Zhang et al., 2015) (Supplementary Table 2). We collected 15 strains with completed and annotated genome sequences (three *P. copri*, two *P. histicola*, four *B. dentium*, and six *B. bifidum)* from the NCBI database (Supplementary Table 3). The 15 genome sequences were analyzed using Vaxign (Figure 3).

In the group of *Bifidobacterium*, *B. dentium ATCC 27679* was used as a seed genome (Figure 3). Out of a total of 2,210 proteins, 1,854 proteins were conserved among the four enriched *B. dentium* genomes, in which 715 proteins were predicted no homology among the six depleted *B. bifidum* genomes. These 715 proteins were then reduced to 152 proteins based on the criteria that they were localized in either outer membrane (OMPs), extracellular, or unknown. Among these 152 proteins, nine proteins were potential auto-antigen, while 143 proteins had no homology to human proteins. Adhesins are critical for pathogens to invade host cells. Therefore, a second filter was applied to select proteins with adhesin probabilities ≥ 0.51. Out of the nine potential auto-antigen, three proteins were predicted to be adhesins. On the other hand, among the 143 no human homology proteins, 77 were predicted to be adhesins.

In the group of *Prevotella*, *P. copri* DSM 1820 was used as a seed genome, and the same workflow described in the previous paragraph was applied (Figure 3). From the analysis, Vaxign identified 83 proteins that were surface-exposed, extracellular, or with unknown location. Among these 83 proteins, two proteins were found to be likely adhesins and auto-antigens, while 17 proteins were predicted to be adhesins with no human homology.

### Identification and Evaluation of 21 Auto-Antigens that Are Potentially Critical for RA Pathogenicity

In this study, we found a total of 21 bacterial proteins in the pathogenic strains (*P. copri* and *B. dentium*) had homology to human proteins through the RM strategy (Table 1). Their mapped human homologous proteins were also identified, and some proteins were found to be related to human diseases (detailed below). Based on the adhesin probabilities (Table 1), five of these auto-antigens, EFM41406.1 (PulA), EFM42429.1 (FabG), EFM42457.1, EFB36361.1, and EFB33962.1 were adhesins, suggesting that these proteins might associate with the host invasion of the bacteria, and therefore, were likely virulence factors.

**TABLE 1:** Vaxign predicted gut microbiome proteins with human homology, and therefore, were likely auto-antigens in human diseases.

**RefSeq (symbol)**	**Adhesin**	**Protein name**	Disease* **and PubMed refs.**
***B. dentium* proteins**			
EFM40687.1	0.275	Glycosyl hydrolase family 2, sugar binding domain protein	Rheumatoid arthritis (1943985; 8480143)
EFM41406.1 (pulA)	0.592	pullulanase, type I	Sjögren’s syndrome (10202179)
EFM40764.1 (thiF)	0.231	thiamine biosynthesis protein ThiF	Dermatomyositis (17763420; 26424665)
EFM42429.1 (fabG)	0.703	oxidoreductase, short chain dehydrogenase/reductase family protein	
EFM42029.1 (ucpA)	0.274	oxidoreductase, short chain dehydrogenase/reductase family protein	
EFM41319.1 (aroK)	0.284	shikimate kinase	
EFM42457.1	0.602	low molecular weight phosphotyrosine protein phosphatase	
EFM40854.1 (ansA)	0.269	L-asparaginase, type I	
EFM40443.1 (xylQ)	0.396	glycosyl hydrolase, family 31	
***P. copri* proteins**			
EFB35607.1	0.360	glycosyl hydrolase family 2, sugar binding domain protein	Rheumatoid arthritis (1943985; 8480143)
EFB36223.1	0.197	nucleotide sugar dehydrogenase	osteoarthritis (25465897)
EFB35842.1	0.415	peptidase, S9A/B/C family, catalytic domain protein	Rheumatoid arthritis (16507127; 25600705)
EFB35735.1	0.272	DnaJ domain protein	Rheumatoid arthritis (23408083)
EFB33829.1 (trxA)	0.302	thioredoxin	Rheumatoid arthritis (28914370; 10384135)
EFB35343.1	0.296	glycosyl hydrolase, family 31	
EFB36361.1	0.870	aldose 1-epimerase	
EFB35772.1	0.260	aconitate hydratase	
EFB35550.1	0.463	putative pyrroline-5-carboxylate reductase	
EFB34687.1	0.443	alpha amylase, catalytic domain protein	
EFB33962.1	0.713	hypothetical protein PREVCOP_06527	
EFB33715.1	0.320	oxidoreductase, short chain dehydrogenase/reductase family protein	

*Disease where the bacterial homologous protein to human is confirmed as an auto-antigen for the disease. The PubMed paper reference with PMID is also provided. The detailed information can be found in Supplementary Table 4.

To further evaluate our predictions, we compared our results with existing literature reports. Among the 21 bacterial proteins with human homology, EFM40687.1, EFB35607.1, EFB35842.1, EFB35735.1, and EFB33829.1 were homologous to four human proteins that were reported to associate with RA (Table 1, Supplementary Table 4). EFM40687.1 and EFB35607.1 had homology to the human protein beta-glucuronidase precursor1-4/X1-X6. The beta-glucuronidase-activity has been found to correlate with histomorphological changes in active arthritis, and the serum activity of beta-glucuronidase indicates the disease activity in RA (Falkenbach et al., 1991). EFB35842.1 has homology to human protein fibroblast activation protein (FAP). FAP plays a key role in the cartilage turnover prevalent in arthritic diseases and promotes proteoglycan loss and subsequently cartilage degradation in RA (Milner et al., 2006). EFB35735.1 has homology to human protein Hsp40. The Hsp40 protein family of bacteria and human origin is suspected to be involved in the pathogenesis of RA, and the sera of RA patients was found to have increased levels of antibodies against human and bacterial Hsp40s (Kotlarz et al., 2013). Finally, EFB33829.1 has homology to human proteins thioredoxin. Thioredoxin concentration in RA patients might be involved in the aggravation of rheumatoid inflammation by augmenting the NF-kappa B activation pathway, and Thioredoxin 1 is associated with the proliferation and apoptosis of RA fibroblast-like synoviocytes (Lu et al., 2018). In summary, all these five predicted autoantigens were verified based on our literature study and were reported to be involved in RA or other rheumatic diseases, and confirmed the utility of our RM method. The other 16 auto-antigens not reported in the literature can also be hypothesized to participate in RA pathogenesis, and in particular, three proteins EFM41406.1, EFM40764.1, and EFB36223.1 were associated with Sjögren’s syndrome, dermatomyositis, and osteoarthritis, respectively (Table 1). These human homology proteins might stimulate autoimmune inflammatory responses, causing autoimmunity and histomorphological damage in RA patients.

### Identification and Evaluation of 94 Microbial Adhesive Proteins, Including 24 ABC Transporters, as Potential RA Virulence Factors

Our Vaxign analysis predicted 94 non-human homology adhesin proteins that were likely associated with RA. These proteins included 24 ATP-binding cassettes (ABC) transporter protein family proteins, 21 hypothetical proteins, 7 LPXTG-motif cell wall anchor domain proteins, 4 glycosyl hydrolase family proteins, 7 DNA-binding proteins, 3 cell wall-binding repeat proteins, 2 receptor family ligand-binding proteins, and 26 other proteins (Table 2, Supplementary Table 5). Among these proteins, 85 proteins had a predicted protegenicity score of over 90%, suggesting that they were highly immunogenic (Supplementary Table 5).

**TABLE 2:** Identified gut microbial adhesin genes as possible causal factors of RA.

**Protein name**	**Localization**	**Human homology**
***B. dentium* proteins**		
ABC transporter protein (24)	EX* (1); UN* (23)	No (24)
glycosyl hydrolase family protein(6)	EX (1); UN (5)	Yes (2); No (4)
LPXTG-motif cell wall anchor domain protein(7)	UN (7)	No (7)
cell wall-binding repeat protein (3)	EX (2); UN (1)	No (3)
DNA-binding protein (2)	UN (2)	No (2)
Receptor family ligand-binding protein (2)	UN (2)	No (2)
hypothetical protein (16)	EX(1); UN (15)	No (16)
Other protein (26)	EX (3); UN (23)	Yes (7); No (19)
***P. copri* proteins**		
glycosyl hydrolase family protein(2)	UN (2)	Yes (2)
DNA-binding protein (5)	UN (5)	No (5)
hypothetical protein (6)	OM*(1); UN (5)	Yes (1)No (5)
Other protein (16)	OM(1); EX (1); UN (14)	Yes (9); No (7)

*EX, Extracellular; UN, Unknown; OM, OuterMembrane.

Among these proteins, 17 proteins came from the *Prevotella copri*, in which 14 proteins were predicted to have protegenicity scores of over 90% (Supplementary Table 5). Studies showed that HLA-DR-presented peptide (T cell epitope) from a 27-kD protein of *P. copri* could stimulate Th1 responses in 42% of RA patients (Pianta et al., 2017). Pianta et al. also reported that *P. copri* induced antibody responses against this protein in RA patients.

In addition, our study identified 24 adhesin ABC transporter proteins and 2 receptor family ligand-binding proteins which had homology to ABC transporter proteins (Supplementary Table 6). We further performed a systematic analysis on the ABC transporter proteins to evaluate our prediction. The ABC transporter protein family is one of the largest superfamilies and is commonly found in bacteria, archaea, and eukaryotes. In humans, over 20 ABC transporters, covering all sub-families, have been found to associate with human diseases (Theodoulou and Kerr, 2015). Several ABC transporters also play an important role in drug metabolism and resistance (Atisha-Fregoso et al., 2016). The role of eukaryotic ABC transporters in RA has been studied for almost two decades (Marki-Zay et al., 2013). Many studies show that eukaryotic ABC transporters associate with drug resistance, disease activity, and progressive destructive arthritis with extra-articular involvement in RA (Atisha-Fregoso et al., 2016; Pascual-Ramos et al., 2016). P-glycoprotein (P-gp), a member of the eukaryotic ABC transporter superfamily, can stimulate CXCR4-overexpressing B cells, and produce rheumatoid factor and various inflammatory cytokines, such as tumor necrosis factor (TNF) and interleukin (IL)-6, and present antigens to T cells in RA (Tsujimura et al., 2018). Antibiotics-like rifampin can also induce the expression of P-gp on specific eukaryotic cells (Hasanuzzaman et al., 2019). Multidrug resistance (MDR)-ABC transporters are widely expressed in cell types relevant to RA pathogenesis and have been used as RA markers (Marki-Zay et al., 2013). All these 26 bacterial ABC transporter proteins (Supplementary Table 6) had predicted protegenicity scores over 94%, demonstrating that they were likely capable to elicit a strong immune response, and thus, might associate with RA pathogenesis.

Although the interactions between eukaryotic ABC transporters and RA are extensively studied as described above, how bacterial ABC transporters are related to RA remains unclear. The ABC transporters are associated with adherence and attachment to host cells and transport an array of substrates, including transition metals, peptides, and amino acids (Garmory and Titball, 2004; Tanaka et al., 2018). For example, the Yfe and Ybt ABC transporter systems *of Yersinia pestis* play a key role for the uptake of inorganic iron (Fetherston et al., 1999; Kirillina et al., 2006). The *ybtP* mutant of *Y. pestis* showed a reduction of iron accumulation and loss of virulence (Fetherston et al., 1999). Many other bacterial ABC transporters also exist, such as the FeoABC transporter of iron and the SitABCD transporter of iron and manganese in *Salmonella enterica* serovar Typhimurium (Boyer et al., 2002). *Staphylococcus aureus* contains ABC transporters that transfer oligopeptides and contribute to bacterial virulence (Garmory and Titball, 2004). GlnQ is a glutamine ABC transporter ATP-binding protein. The *glnQ* mutant of group B *streptococcus* showed decreased adherence and invasion to the host cells and decreased virulence *in vivo* (Tamura et al., 2002). Based on our findings and literature survey, we propose an “ABC-RA hypothesis,” which states that the bacterial ABC transporters are likely involved in RA pathogenesis, possibly through their involvement in bacterial metal or peptide transport, and adherence and attachment to host cells and gut/dental mucosa. These ABC transporters are likely virulence factors by themselves and may stimulate strong protective responses (then as possible vaccine candidates). It is also possible that eukaryotic and bacterial ABC transporters interact with each other. Further experimental analysis is deserved to confirm the function of bacterial ABC transporters in the RA pathogenesis.

In addition to the evaluation of ABC transporters, we also evaluated many other potential virulence factors from our list of 94 predicted proteins (Table 2, Supplementary Tables 5, 6). For example, our study found 7 LPXTG-motif cell wall anchor domain proteins (Table 2). The LPXTG (Leu-Pro-any-Thr-Gly) motif is conserved in over 100 surface proteins of Gram-positive pathogens, such as protein A of *Staphylococcus aureus*, M proteins of *Streptococcus pyogenes*, and several internalins of *Listeria monocytogenes* (Navarre and Schneewind, 1999; Ton-That et al., 1999). Many of these surface proteins and their substrates have experimentally verified roles in pathogenesis (Navarre and Schneewind, 1999; Bubeck Wardenburg et al., 2007), with their involvements in the attachment to specific host tissues during infection (Mazmanian et al., 2002) and evasion of the host immune responses (Schneewind et al., 1993). Such surface proteins can also be applied for vaccine development (Bubeck Wardenburg and Schneewind, 2008). As another example, we found EFB36849.1 (protein name: PREVCOP_03646) as a SusC/RagA family TonB-linked outer membrane protein. SusC/RagA family proteins are likely to import large degradation products of proteins or carbohydrates, and they have been associated with Crohn’s disease and inflammatory bowel disease (Wei et al., 2001). TonB-linked RagA has been validated as a virulence factor of *P. gingivalis* tissue damage and *in vivo* survival (Curtis et al., 1999). TonB-dependent proteins are likely pathogenesis factors (Koebnik, 2005).

## Discussion

This paper has three major contributions in the area of microbiota research. First, we systematically introduce our newly developed Reverse Microbiomics prediction system and describe how RM was used to predict pathogenic antigens of rheumatic diseases in the dysbiotic gut microbiota. Second, RM was applied to predict virulence factors of gut microbiota, and explore the underlying pathogenic mechanism of RA. Third, this study, for the first time, applied an ontology-based literature mining method to systematically collect, represent, and analyze the interaction of gut microbiome associated with RA. We found 23 valuable bacterial species through the ontology-based literature mining method. We also predicted 94 potential virulence factors and 21 potential auto-antigens relevant to RA through the RM pipeline. Our use cases demonstrate that RM was able to predict specific pathogenic factors and auto-antigens of microbiome-associated diseases in dysbiosis microbiota.

To our best knowledge, our RM strategy is the first method for predicting potential virulence factors and protective antigens using reverse bioinformatics analysis on microbiome genomes. A method similar to RM is the Metagenome-wide association studies (MWAS), which focuses on metagenomics shotgun sequencing and identification of the associations between microbiomes and diseases (Wang and Jia, 2016). Being “Reverse,” our RM method starts from the literature mining of experimentally verified knowledge of up- or down-regulated bacteria under different conditions, then ontological classification and analysis of these bacteria, leading to the selection of two pairs of enriched or depleted bacteria for further bioinformatics analysis. Our comparative genomics analysis then reverse-predicts molecular factors and mechanisms that could explain the differential bacterial alteration in patients with a specific microbiome-associated disease. In this study, our prior knowledge was obtained from experimental evidence based on various 16S RNA sequencing and MWAS studies; therefore, our RM study has incorporated the results from the 16S RNA sequencing and MWAS studies.

Another method similar to RM is the reverse ecology, which aims to use a systematic approach to study microbiome ecology and the interactions among different microbes and the environment in a particular microbiome niche. Several network-based reverse ecology approaches, including NetSeed (Carr and Borenstein, 2012), NetCooperate (Levy et al., 2015), RevEcoR (Cao et al., 2016), and PopCOGenT (Arevalo et al., 2019), have been developed to study the interface between species and their environments, and predict the interactions between species. Different from the reverse ecology method, our RM strategy focuses on the rapid bioinformatic prediction of virulence factors (including auto-antigens) contributing to microbiome-associated diseases and protective antigens for potential vaccine and probiotics application. Our RM method is rooted in the Vaxign RV platform that our research group has developed over the past decade. Our RM strategy addresses the challenge of better understanding the fundamental gene-level molecular mechanisms of microbiome-related diseases.

Ontology plays an important role in the standard representation of various data, metadata, and knowledge. The Ontology of Host-Microbiome Interactions (OHMI) developed by our group was used to systematically represent these diseases and microbes associated with rheumatic diseases (He et al., 2019). OHMI provides a consistent and hierarchical representation of the known host-microbiome interactions, where the individual microbes, anatomic locations, host species, and host qualities are also represented using the ontology. As shown in the RA disease use case, we were able to identify those enriched and depleted microbes associated with RA. The ontological classification of enriched microbes suggested possible virulent factors and potential pathogenesis in RA, whereas queries identifying depleted bacteria might identify potential candidates for treatment with biological agents.

For our RA study, both genetic and environmental predisposing factors are involved in the etiopathogenesis (Vaahtovuo et al., 2008). Multiple pieces of evidence have shown that changes in the normal gastrointestinal microflora and dysregulation of the mucosal immune response to these pathogens may help in the development of autoimmune diseases such as RA (Breban, 2016; Mankia and Emery, 2016). The seepage of specific antigen from intestines to circulation and further to joints would lead to chronic articular inflammation observed in RA. The gut microbiota is also a potential new territory for drug targeting (Jia et al., 2008). The molecular mimicry or the cross-reactivity between self-epitopes and pathogen epitopes has been found as a common reason for many pathogen-induced autoimmune diseases. Many pathogens, such as *Klebsiella pneumoniae*, *Proteus mirabilis*, and Lyme disease spirochete *Borrelia burgdorferi* carry antigens that cross-react with human antigens (Weber et al., 2009). The identification of disease-relevant infectious or self-antigens has been challenging in any autoimmune disease, but current RM methods offer innovative approaches to this problem.

To demonstrate the capability of RM application for predicting potential virulence factors of dysbiosis microbiota, we used RM to analyze the genomes of two pairs of altered bacterial species associated with RA: one pair being Gram-positive *B. bifidum* and *B. dentium*, and the other pair being Gram-negative *P. copri* and *P. histicola*. Our current study analyzed 15 complete genomes of the four bacterial species and predicted 115 proteins as potential virulence factors of RA (Figure 3). Among these proteins, we found 21 adhesin proteins in the pathogenic strains of *P. copri* and *B. dentium* and had homology to human proteins. Our literature survey confirmed that five out of these 21 bacterial proteins have homology to four known human auto-antigens (i.e., beta-glucuronidase precursor1-4/X1-X6, FAP, Hsp40, and Thioredoxin) which play important pathological roles in RA (Table 1). There were three bacterial proteins also reported being associated with other inflammatory rheumatic diseases. The results confirmed the ability of RM to predict disease-related factors. Our RM analysis also predicted 94 adhesin proteins with no human homology (Figure 3) likely to be associated with RA. Based on our functional analysis of these proteins, we propose an “ABC-RA hypothesis” that hypothesizes the causal association between ABC transporters and RA formation. Other RA related virulence factors include the LPXTG-motif cell wall anchor domain protein and a SusC/RagA family TonB-linked outer membrane protein. While we made many predictions, our hypotheses based on our auto-antigen and virulence factor predictions are subjected to experimental verification.

For the implementation of the RM strategy, we adopted the freely available Vaxign web application tool. Vaxign was originally designed for RV with a goal for vaccine design, and it has also been applied in predicting pathogen virulence factors (Xiang and He, 2013; Ni et al., 2017; Zhu et al., 2019). In this study, we demonstrated that Vaxign could also be applied to support microbiome-based RM analysis. In the future, we plan to further develop a microbiome-focused tool derived from Vaxign.

The RM strategy can also be applied to study other microbiome-associated diseases for the prediction of new molecular level pathogenesis factors. In addition to the RA use case, we have also applied the RM strategy to study colorectal cancer and gastric cancer cases (unpublished work). The predictive results were also insightful. Given the complexity of each use case presentation, this paper only reported only the RA use case as a proof-of-concept study, and we plan to report the other two use cases in the future. Meanwhile, the RM approach is generically applicable for researchers to test their own use cases.

## Conclusion

To address the important need for the identification of bacterial alteration and explore molecular mechanisms involved in disease development, we developed the new Reverse Microbiomics (RM) strategy. We applied the RM strategy to predict pathogenic auto-antigens and virulence factors associated with rheumatoid arthritis (RA) in the dysbiotic gut microbiota. The RM strategy includes the literature mining and annotation, ontology classification of mined results, and reverse informatics analysis inspired by reverse vaccinology. The reverse informatics analysis applies comparative genomics methods to identify new microbial gene factors contributing to the disease pathogenesis. Our strategy was used to study rheumatoid arthritis (RA). A total of 15 genomes from a pair of Gram-positive bacteria (*B. bifidum* and *B. dentium*) and a pair of Gram-negative bacteria (*P. copri* and *P. histicola*) were selected and analyzed using the reverse comparative genome sequence analysis. Our study predicted 21 auto-antigens and 94 non-human homologous proteins, which were likely associated with RA formation. Many of these predictions were verified from published literature reports, and new hypotheses could be generated. The RM strategy presented here is also applicable to other microbiome-associated diseases. Overall, the RM strategy facilitates our understanding of diseases involving intricate host-microbiome interactions from bacterial level association to molecular level mechanism prediction.

## Data Availability Statement

All datasets generated for this study are included in the article/Supplementary Material.

## Author Contributions

HW performed RA knowledge extraction, ontology representation, data analysis, and result interpretation. EO performed Vaxign data analysis. JK provided inflammatory disease and microbiome consultation and result interpretation. DS provided pharmacology and microbiome consultation and result interpretation. YH contributed to project design, ontology development, and data analysis. HW and YH contributed to the manuscript preparation and discussion. All authors contributed to the article and approved the submitted version.

## Conflict of Interest

The authors declare that the research was conducted in the absence of any commercial or financial relationships that could be construed as a potential conflict of interest.

## Supplementary Material

Supplementary Table 1 Summary of literature on gut microbiome in rheumatoid arthritis.

Supplementary Table 2 The microbiome bacteria are associated with RA.

Supplementary Table 3 15 complete gut microbiome genomes used in our Reverse Microbiomics study.

Supplementary Table 4 The human proteins that have homology with *B. dentium* and *P. copri* protein.

Supplementary Table 5 Vaxign predicted gut microbiome no human homology proteins.

Supplementary Table 6 24 ABC transporter adhesin proteins and two related proteins in *B. dentium* but not present in *B. bifidum.*
