# Supplementary material for: Reverse Microbiomics: A New Reverse Dysbiosis Analysis Strategy and Its Usage in Prediction of Autoantigens and Virulent Factors in Dysbiotic Gut Microbiomes From Rheumatoid Arthritis Patients
Source: Front Microbiol. 2021 Feb 25;12:633732. doi: 10.3389/fmicb.2021.633732 (PMC7947680; doi:10.3389/fmicb.2021.633732)
Supplement: Supplementary Table 2 — The microbiome bacteria are associated with RA. [file Table_2.docx]

**Supplementary Table 2 The microbiome bacteria are associated with RA.**

| **No.** | **Scientific name** | **Taxon ID** | **Rank** | **Alterations** | **Localization** | **PMID** |
| --- | --- | --- | --- | --- | --- | --- |
| 1 | *Bacteroides fragilis* | 817 | species | Decrease | gut | 18528968 |
| 2 | *Bacteroides plebeius* | 310297 | species | Decrease | gut | 26214836 |
| 3 | *Bifidobacterium bifidum* | 1681 | species | Decrease | gut | 26214836 |
| 4 | *Haemophilus sp.* | 740 | species | Decrease | oral/gut | 26214836 |
| 5 | *Klebsiella pneumoniae* | 573 | species | Decrease | gut | 26214836 |
| 6 | *Megamonas hypermegale* | 158847 | species | Decrease | gut | 26214836 |
| 7 | *Prevotella histicola* | 470565 | species | Decrease | gut | 28598360 |
| 8 | *Sutterella wadsworthensis* | 40545 | species | Decrease | gut | 26214836 |
| 9 | *Lactobacillus casei* | 1582 | species | Probiotic | gut | 22749779; 24673738 |
| 10 | *Eggerthella lenta* | 84112 | species | Increase | gut | 26214836 |
| 11 | *Escherichia coli* | 562 | species | Increase | gut | 20360042; 16091399 |
| 12 | *Gordonibacter pamelaeae* | 471189 | species | Increase | gut | 26214836 |
| 13 | *Bacteroides sp.* | 29523 | species | Increase | gut | 26214836 |
| 14 | *Bifidobacterium dentium* | 1689 | species | Increase | gut | 26214836 |
| 15 | *Lachnospiraceae bacterium* | 1898203 | species | Increase | gut | 26214836 |
| 16 | *Collinsella aerofaciens* | 74426 | species | Increase | gut | 27102666 |
| 17 | *Lactobacillus salivarius* | 1624 | species | Increase | oral/gut | 26214836; 27789760 |
| 18 | *Lactobacillus sp* | 1591 | species | Increase | gut | 26214836 |
| 19 | *Proteus mirabilis* | 584 | species | Increase | gut/ urine | 19895906; 24873878;  9032817 |
| 20 | *Prevotella copri* | 165179 | species | Increase | gut | 27333153 |
| 21 | *Clostridium asparagiforme* | 333367 | species | Increase | gut | 26214836 |
| 22 | *Ruminococcus lactaris* | 46228 | species | Increase | gut | 26214836 |
| 23 | *Clostridium perfringens* | 1502 | species | Increase | gut | 2889500 |
| 24 | *Veillonella* | 29465 | genus | Decrease | gut/oral | 26214836 |
| 25 | *Porphyromonas* | 836 | genus | Decrease | gut/ airway | 18528968;  27855721 |
| 26 | *Prevotella* | 838 | genus | Decrease | gut/ airway | 26385261;  27855721 |
| 27 | *Blautia coccoides* | 1532 | genus | Decrease | gut | 18528968 |
| 28 | *Eggerthella* | 84111 | genus | Increase | gut | 28376066 |
| 29 | *Faecalibacterium* | 216851 | genus | Decrease | gut | 27333153 |
| 30 | *Bacteroides* | 816 | genus | Decrease | gut | 26385261 |
| 31 | *Prevotellaceae* | 171552 | family | Increase | gut | 24192039 |
| 32 | *Clostridia* | 186802 | order | Increase | gut | 26385261; 2889500 |
| 33 | *Actinobacteria* | 201174 | phylum | Increase | gut | 28376066 |
| 34 | *Neisseria sp.* | 192066 | species | Decrease | oral | 26214836 |
| 35 | *Eikenella* | 538 | genus | Decrease | oral | 26214836 |
| 36 | *Cryptobacterium curtum* | 84163 | species | Increase | oral | 26214836 |
| 37 | *Aggregatibacter* | 416916 | genus | Decrease | oral | 26214836 |
| 38 | *Atopobium sp.* | 1872650 | species | Increase | oral | 26214836 |
| 39 | *Actinomyces* | 1654 | genus | Increase | oral | 26214836; |
| 40 | *Cardiobacterium* | 2717 | genus | Decrease | oral | 26214836 |
| 41 | *Haemophilus* | 724 | genus | Decrease | oral | 26214836 |
| 42 | *Kingella* | 32257 | genus | Decrease | oral | 26214836 |
| 43 | *Leptotrichia sp.* | 104608 | species | Increase | oral | 22576262 |
| 44 | *Porphyromonas gingivalis* | 837 | species | Increase | oral | 19554393; 23902301; 27641915; 27789760  20436074; 24873878 |
| 45 | *Prevotella* | 838 | genus | Increase | oral | 22576262 |
| 46 | *Prevotella intermedia* | 28131 | species | Increase | oral | 22576262; 27789760 |
| 47 | *Rothia aeria* | 172042 | species | Decrease | oral | 26214836 |
| 48 | *Tannerella forsythia* | 28112 | species | Increase | oral | 19554393; 22576262; 23902301 |
| 49 | *Treponema* | 157 | genus | Decrease | airway | 27855721 |
| 50 | *Pseudonocardia* | 1847 | genus | Increase | airway | 27855721 |
| 51 | *Burkholderia* | 32008 | genus | Decrease | airway | 27855721 |
| 52 | *Methylobacterium* | 407 | genus | Increase | airway | 27855721 |
| 53 | *Micrococcus* | 1269 | genus | Increase | airway | 27855721 |
| 54 | *Actinomyces* | 1654 | genus | Decrease | airway | 27855721 |
