# Supplementary material for: Reverse Microbiomics: A New Reverse Dysbiosis Analysis Strategy and Its Usage in Prediction of Autoantigens and Virulent Factors in Dysbiotic Gut Microbiomes From Rheumatoid Arthritis Patients
Source: Front Microbiol. 2021 Feb 25;12:633732. doi: 10.3389/fmicb.2021.633732 (PMC7947680; doi:10.3389/fmicb.2021.633732)
Supplement: Supplementary Table 3 — 15 complete gut microbiome genomes used in our Reverse Microbiomics study. [file Table_3.docx]

|  |
| --- |

**Supplementary Table 3 15 complete gut microbiome genomes used in our Reverse Microbiomics study.**

| NO. | **strain** | **NCBI Bio Project No.** | **Proteins number** | **Alterations in gut** |
| --- | --- | --- | --- | --- |
| **Species:** ***Bifidobacterium dentium*** | | | | |
| 1 | ATCC 27679 | PRJNA51373 | 2210 | Up-regulation |
| 2 | ATCC 27678 | PRJNA20555 | 2149 | Up-regulation |
| 3 | Bd1 | PRJNA17583 | 2137 | Up-regulation |
| 4 | JCVIHMP022 | PRJNA31395 | 2107 | Up-regulation |
| **Species: *Bifidobacterium bifidum*** | | | | |
| 5 | BGN4 | PRJNA21077 | 1835 |  |
| 6 | NCIMB41171 | PRJNA30055 | 1738 | Down-regulation |
| 7 | PRL2010 | PRJNA42863 | 1706 | Down-regulation |
| 8 | S17 | PRJNA51963 | 1715 | Down-regulation |
| 9 | BF3 | PRJNA270902 | 1696 | Down-regulation |
| 10 | MJR8628B | PRJNA272088 | 1814 | Down-regulation |
| **Species: *Prevotella copri*** | | | | |
| 11 | DSM 18205 | PRJNA30025 | 2995 | Up-regulation |
| 12 | Indica | PRJNA393007 | 3271 | Up-regulation |
| 13 | AF38-11 | PRJNA482748 | 2840 | Up-regulation |
| **Species: *Prevotella histicola*** | | | | |
| 14 | F0411 | PRJNA49883 | 2277 | Down-regulation |
| 15 | JCM 15637 | PRJNA219666 | 2400 | Down-regulation |
