# Supplementary material for: Reverse Microbiomics: A New Reverse Dysbiosis Analysis Strategy and Its Usage in Prediction of Autoantigens and Virulent Factors in Dysbiotic Gut Microbiomes From Rheumatoid Arthritis Patients
Source: Front Microbiol. 2021 Feb 25;12:633732. doi: 10.3389/fmicb.2021.633732 (PMC7947680; doi:10.3389/fmicb.2021.633732)
Supplement: Supplementary Table 4 — The human proteins that have homology with B. dentium and P. copri protein. [file Table_4.docx]

**Supplementary Table 4 The human proteins that have homology with *B. dentium* and *P. copri* protein.**

| **No.** | **Bacterium** | **protein ID** | **Protein name** | **Human protein name** | **comments** | **PubMed IDs** |
| --- | --- | --- | --- | --- | --- | --- |
| 1 | ***B. dentium*** | EFM41406.1 | pullulanase, type I | alpha-amylase | Alpha-amylase functions as a salivary gland-specific self T cell epitope in patients with Sjögren's syndrome | 10202179 |
| 2 | ***B. dentium*** | EFM41319.1 | shikimate kinase | 1.probable gluconokinase isoform 1 /3/X1/X2.X3/X4/X6  2.interferon regulatory factor 2 binding protein | interferon regulatory factor 2 binding protein 2b is a negative regulator of the nuclear factor of activated T cells (NFAT1)transcription factor | 21576369 |
| 3 | ***B. dentium*** | EFM40687.1 | Glycosyl hydrolase family 2, sugar binding domain protein | beta-glucuronidase precursor1-4/X1-X6 | beta-glucuronidase-activity has been found to correlate well with histomorphological changes in active arthritis.The serum activity of beta-glucuronidase indicate the disease activity in rheumatoid arthritis (RA) | 1943985;  8480143 |
| 4 | ***B. dentium*** | EFM40764.1 | thiamine biosynthesis protein ThiF | 1.adenylyltransferase and sulfurtransferase MOCS3  2.Chain B, Ubiquitin-like 2 Activating Enzyme E1b  3.NEDD8-activating enzyme E1 catalytic subunit isoform 1  4. UBA3  5. SUMO-1-activating enzyme E1 C subunit | anti-small ubiquitin-like modifier-1 (SUMO-1) activating enzyme (SAE) is a new dermatomyositis (DM)-specific autoantibodies | 17763420;  26424665 |
| 5 | ***B. dentium*** | EFM40443.1 | glycosyl hydrolase, family 31 | 1.neutral alpha-glucosidase C type 3  2.glucosidase  3.maltase-glucoamylase |  | No |
| 6 | ***B. dentium*** | EFM42429.1 | oxidoreductase, short chain  dehydrogenase/reductase family protein | 1.HPDHase  2.peroxisomal trans-2-enoyl-CoA reductase  3.Hep27-like protein  4.HSD17B8  5.Chain A, Dehydrogenase/reductase SDR family member 4  6.Chain A, 17-beta-hydroxysteroid dehydrogenase 14 |  | No |
| 7 | ***B. dentium*** | EFM42029.1 | oxidoreductase, short chain dehydrogenase/reductase family protein | 1.Hep27-like protein  2.Chain A, Dehydrogenase/reductase SDR family member 4  3.NADPH-dependent retinol dehydrogenase/ reductase  4.dehydrogenase/reductase SDR family member 4 isoform 1  5.HPDHase |  | No |
| 8 | ***B. dentium*** | EFM42457.1 | low molecular weight phosphotyrosine protein phosphatase | 1. adipocyte acid phosphatase beta  2. cytoplasmic phosphotyrosyl protein phosphatase  3. zonadhesin isoform 3 precursor |  | No |
| 9 | ***B. dentium*** | EFM40854.1 | L-asparaginase, type I | 1. ASPG protein  2. hCG23215, isoform CRA_a  3.60 kDa lysophospholipase isoform X1-13 |  | No |
| 10 | ***P. copri*** | EFB35607.1 | glycosyl hydrolase family 2, sugar binding domain protein | 1. Chain A, Beta-glucuronidase  2. beta-glucuronidase isoform 1-4/X1-X6 | beta-glucuronidase-activity has been found to correlate well with histomorphological changes in active arthritis. The serum activity of beta-glucuronidase indicate the disease activity in rheumatoid arthritis (RA) | 1943985;  8480143 |
| 11 | ***P. copri*** | EFB36361.1 | aldose 1-epimerase | 1. aldose 1-epimerase  2. Chain A, Aldose 1-epimerase  3. aldose 1-epimerase isoform X1 |  | No |
| 12 | ***P. copri*** | EFB36223.1 | nucleotide sugar dehydrogenase | 1. UDP-glucose dehydrogenase, isoform CRA_c  2. Chain A, Udp-glucose 6-dehydrogenase | UDP-glucose dehydrogenase modulates proteoglycan synthesis in articular chondrocytes | 25465897 |
| 13 | ***P. copri*** | EFB35842.1 | peptidase, S9A/B/C family,  catalytic domain protein | 1. prolyl endopeptidase FAP isoform 2  2. fibroblast activation protein  3. cytoplasmic Seprase truncated isoform  4. Chain A, Dipeptidyl Peptidase | 1. Fibroblast activation protein (FAP) alpha play an important pathological role in the cartilage turnover prevalent in arthritic diseases.  2. Fibroblast activation protein (FAP) is high expression protein, and FAP promote proteoglycan loss and subsequently cartilage degradation in rheumatoid arthritis (RA). | 16507127;  25600705 |
| 14 | ***P. copri*** | EFB35735.1 | DnaJ domain protein | 1. dnaJ homolog subfamily B member 5 isoform 1-4  2. DnaJ (Hsp40) homolog, subfamily A, member 1, isoform CRA_a  3. DnaJA2 | Hsp40 could be the targets of antibodies originally directed against bacterial DnaJ in RA | 23408083 |
| 15 | ***P. copri*** | EFB33829.1 | thioredoxin | 1. Chain A, Thioredoxin  2. Chain B, Thioredoxin 1  3. thioredoxin  4. thioredoxin isoform 1 | 1. Thioredoxin 1 is associated with the proliferation and apoptosis of rheumatoid arthritis fibroblast-like synoviocytes.  2. Thioredoxin concentration in RA patients might be involved in the aggravation of rheumatoid inflammation by augmenting the NF-kappaB activation pathway. | 28914370;  10384135 |
| 16 | ***P. copri*** | EFB35772.1 | aconitate hydratase | 1. Aconitase 2, mitochondrial  2. ACO2  3. aconitate hydratase, mitochondrial precursor  4. iron-responsive regulatory protein/iron regulatory protein 1 |  | No |
| 17 | ***P. copri*** | EFB35550.1 | putative pyrroline-5-carboxylate reductase | 1. pyrroline-5-carboxylate reductase 3 isoform 1  2.pyrroline-5-carboxylate reductase-like, isoform CRA_b  3. pyrroline-5-carboxylate reductase 1 |  | No |
| 18 | ***P. copri*** | EFB34687.1 | alpha amylase, catalytic domain protein | 1. SLC3A1 variant C  2. amino acid transport protein  3. neutral and basic amino acid transport protein rBAT  4. 4F2 cell-surface antigen heavy chain isoform c  5. lymphocyte activation antigen |  | No |
| 19 | ***P. copri*** | EFB33962.1 | hypothetical protein PREVCOP_06527 | sialate O-acetylesterase isoform 1/2/X1 |  | No |
| 20 | ***P. copri*** | EFB35343.1 | glycosyl hydrolase, family 31 | 1. Sneutral alpha-glucosidase AB isoform 2- 7  2. glucosidase |  | No |
| 21 | ***P. copri*** | EFB33715.1 | oxidoreductase, short chain  dehydrogenase/reductase family protein | 1. Chain A, Corticosteroid 11-beta-dehydrogenase isozyme 1  2. very-long-chain 3-oxoacyl-CoA reductase  3.15-hydroxyprostaglandin dehydrogenase [NAD(+)] isoform 1 |  | No |
