# Supplementary material for: Reverse Microbiomics: A New Reverse Dysbiosis Analysis Strategy and Its Usage in Prediction of Autoantigens and Virulent Factors in Dysbiotic Gut Microbiomes From Rheumatoid Arthritis Patients
Source: Front Microbiol. 2021 Feb 25;12:633732. doi: 10.3389/fmicb.2021.633732 (PMC7947680; doi:10.3389/fmicb.2021.633732)
Supplement: Supplementary Table 5 — Vaxign predicted gut microbiome no human homology proteins. [file Table_5.docx]

**Supplementary Table 5 Vaxign predicted gut microbiome no human homology proteins.**

| **No.** | **Protein**  **RefSeq** | **Symbol** | **Localization** | **Adhesin** | **TMH** | **Human**  **homology** | **Protein Name** | **Protegenicity Score (%)** |
| --- | --- | --- | --- | --- | --- | --- | --- | --- |
| ABC transporter protein | | | | | | | | |
| 1 | EFM40667.1 | HMPREF0168_1690 | UN | 0.511 | 0 | No | ABC transporter, solute-binding protein | 99.38 |
| 2 | EFM40440.1 | HMPREF0168_2067 | UN | 0.643 | 0 | No | ABC transporter, solute-binding protein, partial | 96.77 |
| 3 | EFM40447.1 | HMPREF0168_2074 | UN | 0.525 | 0 | No | ABC transporter, solute-binding protein | 98.24 |
| 4 | EFM40528.1 | amyE | UN | 0.699 | 1 | No | ABC transporter, solute-binding protein | 98.47 |
| 5 | EFM40553.1 | HMPREF0168_2034 | UN | 0.583 | 1 | No | ABC transporter, solute-binding protein | 99.24 |
| 6 | EFM40564.1 | HMPREF0168_2045 | UN | 0.585 | 0 | No | ABC transporter, solute-binding protein | 98.42 |
| 7 | EFM40775.1 | HMPREF0168_1798 | UN | 0.589 | 0 | No | ABC transporter, solute-binding protein | 99.13 |
| 8 | EFM40789.1 | HMPREF0168_1812 | UN | 0.658 | 0 | No | ABC transporter, solute-binding protein | 97.66 |
| 9 | EFM40803.1 | HMPREF0168_1826 | UN | 0.639 | 0 | No | ABC transporter, solute-binding protein | 99.08 |
| 10 | EFM42554.1 | HMPREF0168_0043 | UN | 0.638 | 0 | No | ABC transporter, solute-binding protein | 99.56 |
| 11 | EFM41928.1 | dppA2 | UN | 0.549 | 0 | No | ABC transporter, substrate-binding protein, family 5 | 99.86 |
| 12 | EFM41527.1 | HMPREF0168_0920 | UN | 0.590 | 0 | No | ABC transporter, solute-binding protein | 99.40 |
| 13 | EFM40538.1 | HMPREF0168_2019 | UN | 0.537 | 0 | No | ABC transporter, solute-binding protein | 99.86 |
| 14 | EFM40465.1 | HMPREF0168_2092 | UN | 0.548 | 0 | No | ABC transporter, solute-binding protein | 99.79 |
| 15 | EFM41333.1 | HMPREF0168_1649 | EX | 0.531 | 0 | No | ABC transporter, solute-binding protein | 98.03 |
| 16 | EFM41148.1 | HMPREF0168_1464 | UN | 0.629 | 0 | No | ABC transporter, substrate-binding protein, family 3 | 95.12 |
| 17 | EFM40959.1 | HMPREF0168_1275 | UN | 0.671 | 2 | No | ABC transporter permease | 95.35 |
| 18 | EFM41049.1 | HMPREF0168_1365 | UN | 0.573 | 0 | No | sugar ABC transporter substrate-binding protein | 99.38 |
| 19 | EFM41927.1 | dppA | UN | 0.621 | 0 | No | ABC transporter, substrate-binding protein,family5 | 99.86 |
| 20 | EFM41054.1 | HMPREF0168_1370 | UN | 0.624 | 0 | No | ABC transporter, solute-binding protein | 97.55 |
| 21 | EFM41009.1 | HMPREF0168_1325 | UN | 0.713 | 0 | No | ABC transporter, substrate-binding protein,  family 3 | 95.21 |
| 22 | EFM42419.1 | HMPREF0168_0073 | UN | 0.584 | 1 | No | ABC transporter, substrate-binding protein | 96.89 |
| 23 | EFM42117.1 | HMPREF0168_0600 | UN | 0.614 | 1 | No | ABC transporter, solute-binding protein | 99.79 |
| 24 | EFM41129.1 | HMPREF0168_1445 | UN | 0.518 | 0 | No | carbohydrate ABC transporter substrate-binding protein | 94.21 |
| hypothetical protein | | | | | | | | |
| 25 | EFM41857.1 | HMPREF0168_0340 | UN | 0.590 | 0 | No | hypothetical protein HMPREF0168_0340 | 90.91 |
| 26 | EFM41759.1 | HMPREF0168_1152 | UN | 0.618 | 1 | No | hypothetical protein HMPREF0168_1152 | 91.12 |
| 27 | EFM41133.1 | HMPREF0168_1449 | UN | 0.581 | 0 | No | hypothetical protein HMPREF0168_1449 | 74.28 |
| 28 | EFM40281.1 | HMPREF0168_2261 | UN | 0.541 | 1 | No | hypothetical protein HMPREF0168_2261 | 90.91 |
| 29 | EFM42495.1 | HMPREF0168_0149 | UN | 0.519 | 0 | No | hypothetical protein HMPREF0168_0149 | 90.91 |
| 30 | EFM42388.1 | HMPREF0168_0260 | EX | 0.530 | 1 | No | hypothetical protein HMPREF0168_0260 | 34.03 |
| 31 | EFM42456.1 | HMPREF0168_0110 | UN | 0.624 | 1 | No | hypothetical protein HMPREF0168_0110 | 91.28 |
| 32 | EFM42439.1 | HMPREF0168_0093 | UN | 0.532 | 1 | No | hypothetical protein HMPREF0168_0093 | 98.03 |
| 33 | EFM41520.1 | HMPREF0168_0913 | UN | 0.682 | 1 | No | hypothetical protein HMPREF0168_0913 | 90.91 |
| 34 | EFM40975.1 | HMPREF0168_1291 | UN | 0.631 | 0 | No | hypothetical protein HMPREF0168_1291 | 59.47 |
| 35 | EFM42434.1 | HMPREF0168_0088 | UN | 0.678 | 1 | No | hypothetical protein HMPREF0168_0088 | 90.91 |
| 36 | EFM40781.1 | HMPREF0168_1804 | UN | 0.587 | 1 | No | hypothetical protein HMPREF0168_1804 | 90.91 |
| 37 | EFM40306.1 | HMPREF0168_2286 | UN | 0.697 | 1 | No | hypothetical protein HMPREF0168_2286 | 98.51 |
| 38 | EFM40245.1 | HMPREF0168_2337 | UN | 0.594 | 1 | No | hypothetical protein HMPREF0168_2337 | 90.91 |
| 39 | EFM40888.1 | HMPREF0168_1911 | UN | 0.617 | 1 | No | hypothetical protein HMPREF0168_1911 | 90.91 |
| 40 | EFM40837.1 | HMPREF0168_1860 | UN | 0.710 | 0 | No | hypothetical protein HMPREF0168_1860 | 88.46 |
| 41 | EFB36194.1 | PREVCOP_04240 | UN | 0.531 | 1 | No | hypothetical protein PREVCOP_04240 | 77.22 |
| 42 | EFB34862.1 | PREVCOP_05581 | UN | 0.659 | 0 | No | hypothetical protein PREVCOP_05581 | 92.17 |
| 43 | EFB34849.1 | PREVCOP_05679 | OM | 0.827 | 0 | No | hypothetical protein PREVCOP_05679 | 90.91 |
| 44 | EFB34394.1 | PREVCOP_06113 | UN | 0.723 | 0 | No | hypothetical protein PREVCOP_06113 | 90.91 |
| 45 | EFB36891.1 | thiD | UN | 0.714 | 1 |  | phosphomethylpyrimidine kinase | 90.91 |
| LPXTG-motif cell wall anchor domain protein | | | | | | | |  |
| 46 | EFM40548.1 | HMPREF0168_2029 | UN | 0.649 | 2 | No | LPXTG-motif cell wall anchor domain protein | 98.47 |
| 47 | EFM40547.1 | HMPREF0168_2028 | UN | 0.671 | 2 | No | LPXTG-motif cell wall anchor domain protein | 99.89 |
| 48 | EFM40711.1 | HMPREF0168_1734 | UN | 0.660 | 2 | No | LPXTG-motif cell wall anchor domain protein | 98.72 |
| 49 | EFM41082.1 | HMPREF0168_1398 | UN | 0.588 | 2 | No | LPXTG-motif cell wall anchor domain protein | 98.86 |
| 50 | EFM42133.1 | HMPREF0168_0616 | UN | 0.596 | 1 | No | LPXTG-motif cell wall anchor domain protein | 99.52 |
| 51 | EFM40253.1 | HMPREF0168_2328 | UN | 0.848 | 2 | No | LPXTG-motif cell wall anchor domain protein | 97.66 |
| 52 | EFM41956.1 | HMPREF0168_0439 | UN | 0.570 | 1 | No | LPXTG-motif cell wall anchor domain protein | 99.86 |
| glycosyl hydrolase family protein | | | | | | | |  |
| 53 | EFM40689.1 | HMPREF0168_1712 | UN | 0.632 | 2 | No | glycosyl hydrolase family 3 N-terminal domain protein | 99.86 |
| 54 | EFM41107.1 | HMPREF0168_1423 | EX | 0.597 | 2 | No | glycosyl hydrolase family 3 N-terminal domain protein | 99.86 |
| 55 | EFM42316.1 | HMPREF0168_0188 | UN | 0.577 | 0 | No | glycosyl hydrolase | 90.66 |
| 56 | EFM42358.1 | HMPREF0168_0230 | UN | 0.577 | 2 | No | glycosyl hydrolase family 3 N-terminal domain protein | 99.91 |
| cell wall-binding repeat protein | | | | | | | |  |
| 57 | EFM40299.1 | HMPREF0168_2279 | EX | 0.657 | 1 | No | cell wall-binding repeat protein | 90.91 |
| 58 | EFM40291.1 | HMPREF0168_2271 | EX | 0.600 | 1 | No | cell wall-binding repeat protein | 99.89 |
| 59 | EFM40286.1 | amiD2 | UN | 0.597 | 1 | No | cell wall-binding repeat protein | 94.14 |
| DNA-binding protein | | | | | | | | |
| 60 | EFB33709.1 | PREVCOP_06815 | UN | 0.571 | 0 | No | putative DNA-binding protein | 90.91 |
| 61 | EFB35383.1 | PREVCOP_05049 | UN | 0.823 | 0 | No | putative DNA-binding protein | 90.91 |
| 62 | EFB35865.1 | PREVCOP_04770 | UN | 0.553 | 0 | No | putative DNA-binding protein | 90.91 |
| 63 | EFB35978.1 | PREVCOP_04397 | UN | 0.704 | 0 | No | putative DNA-binding protein | 90.91 |
| 64 | EFB36781.1 | PREVCOP_03832 | UN | 0.635 | 0 | No | putative DNA-binding protein | 90.91 |
| 65 | EFM42426.1 | HMPREF0168_0080 | UN | 0.512 | 0 | No | DNA-binding helix-turn-helix protein | 90.91 |
| 66 | EFM41648.1 | vapI | UN | 0.524 | 0 | No | DNA-binding helix-turn-helix protein | 76.14 |
| Receptor family ligand-binding protein | | | | | | | |  |
| 67 | EFM41039.1 | HMPREF0168_1355 | UN | 0.584 | 0 | No | receptor family ligand-binding protein | 99.34 |
| 68 | EFM40815.1 | braC2 | UN | 0.611 | 1 | No | receptor family ligand-binding protein | 99.40 |
| Other protein | | | | | | | |  |
| 69 | EFM41452.1 | HMPREF0168_0845 | UN | 0.575 | 0 | No | phage minor structural protein, N-terminal domain protein | 97.92 |
| 70 | EFM41083.1 | HMPREF0168_1399 | UN | 0.704 | 2 | No | polymorphic outer membrane protein repeat (3 repeats) | 98.47 |
| 71 | EFM40769.1 | HMPREF0168_1792 | EX | 0.638 | 2 | No | conserved repeat protein | 99.95 |
| 72 | EFM40712.1 | HMPREF0168_1735 | UN | 0.676 | 1 | No | pilin isopeptide linkage domain protein | 99.40 |
| 73 | EFM40453.1 | HMPREF0168_2080 | UN | 0.520 | 3 | No | cellulase (glycosyl hydrolase family 5) | 98.90 |
| 74 | EFM40356.1 | HMPREF0168_2166 | UN | 0.611 | 2 | No | histidine acid phosphatase | 98.03 |
| 75 | EFM40340.1 | HMPREF0168_2150 | UN | 0.610 | 2 | No | von Willebrand factor type A domain protein | 99.86 |
| 76 | EFM40289.1 | HMPREF0168_2269 | UN | 0.535 | 1 | No | GDSL-like protein | 90.91 |
| 77 | EFM41610.1 | HMPREF0168_1003 | UN | 0.618 | 1 | No | cell envelope-like function transcriptional attenuator common domain protein | 91.53 |
| 78 | EFM40282.1 | HMPREF0168_2262 | UN | 0.706 | 2 | No | conjugal transfer protein | 91.12 |
| 79 | EFM40731.1 | HMPREF0168_1754 | UN | 0.535 | 1 | No | ethanolamine utilization protein EutL | 90.91 |
| 80 | EFM40283.1 | HMPREF0168_2263 | UN | 0.635 | 0 | No | sialate O-acetylesterase | 95.95 |
| 81 | EFM40849.1 | HMPREF0168_1872 | EX | 0.696 | 1 | No | serine hydrolase | 94.50 |
| 82 | EFM40814.1 | braC | UN | 0.627 | 0 | No | receptor family ligand-binding protein | 98.74 |
| 83 | EFM41833.1 | HMPREF0168_1226 | UN | 0.531 | 0 | No | WXG100 family type VII secretion target | 78.27 |
| 84 | EFM40737.1 | rbsB | UN | 0.562 | 0 | No | sugar-binding domain protein | 96.08 |
| 85 | EFM40333.1 | HMPREF0168_2143 | UN | 0.842 | 0 | No | TQXA domain protein, partial | 92.31 |
| 86 | EFM40285.1 | HMPREF0168_2265 | UN | 0.620 | 0 | No | hypothetical protein HMPREF0168_2265 | 90.91 |
| 87 | EFM40966.1 | metQ | UN | 0.615 | 0 | No | NLPA lipoprotein | 94.92 |
| 88 | EFB36701.1 | PREVCOP_03747 | UN | 0.540 | 0 | No | WD40-like protein | 98.88 |
| 89 | EFB36206.1 | PREVCOP_04252 | UN | 0.690 | 0 | No | conserved hypothetical protein TIGR03905 | 88.46 |
| 90 | EFB35804.1 | PREVCOP_04708 | UN | 0.574 | 2 | No | polysaccharide biosynthesis/export protein | 90.91 |
| 91 | EFB35183.1 | PREVCOP_05317 | UN | 0.522 | 0 | No | carbohydrate binding domain protein | 96.63 |
| 92 | EFB33928.1 | PREVCOP_06599 | UN | 0.522 | 0 | No | single-strand binding family protein | 80.12 |
| 93 | EFB34544.1 | rnfG | UN | 0.631 | 1 | No | electron transport complex, RnfABCDGE type, G subunit | 91.14 |
| 94 | EFB36849.1 | PREVCOP_03646 | OM | 0.877 | 1 | No | TonB-linked outer membrane protein,  SusC/RagA family | 99.89 |

EX: Extracellular;UN: Unknown
